# Predictability of electron cone ratios with respect to linac make and model

**DOI:** 10.1120/jacmp.v4i2.2534

**Published:** 2003-03-01

**Authors:** R. C. Tailor, D. S. Followill, N. Hernandez, G. S. Ibbott, W. F. Hanson

**Affiliations:** ^1^ Department of Radiation Physics The University of Texas M. D. Anderson Cancer Center Houston Texas 77030

**Keywords:** cone ratio, quality assurance, electron dosimetry

## Abstract

In the past, the Radiological Physics Center (RPC) has developed standard sets of photon depth‐dose and wedge‐factor data, specific to the make, model, and wedge design of the linear accelerator (linac). In this paper, the RPC extends the same concept to electron‐cone ratios. Since 1987, the RPC has measured and documented cone‐ratio (CR) values during on‐site dosimetry review visits to institutions participating in National Cancer Institute cooperative clinical trials. Data have been collected for approximately 500 electron beams from a wide spectrum of linac models. The analysis presented in this paper indicates that CR values are predictable to 2% to 3% (two standard deviations) for a given make and model of linac with a few exceptions. The analysis also revealed some other interesting systematics. For some models, such as the Varian Clinac 2500 and the Elekta/Philips SL18, SL20, and SL25, CR values were nearly identical for cone sizes 15 cm×15 cm (or 14 cm×14 cm) and 20 cm×20 cm across the range of available energies. Certain models of the same make of linac, such as the Mevatron MD, KD, and 6700 series models or the Clinac 2100 and 2300 models, exhibited indistinguishable CRs. Irrespective of linac model, two consistent general trends were observed: namely, an increase in CR value with incident beam energy for cone sizes smaller than 10 cm×10 cm and a decrease with energy for cone sizes larger than 10 cm×10 cm. These data are valuable to the RPC as a quality assurance remote‐monitoring tool to identify potential dosimetry errors. The physics community will also find the data useful in several ways: as a redundant check for clinical values in use, to validate the values measured during commissioning of new machines or to ensure consistency of values measured during annual quality assurance procedures.

PACS number(s): 87.54.–n, 87.53.–j

## INTRODUCTION

Since 1969, the Radiological Physics Center (RPC) has conducted on‐site dosimetry review visits to the institutions participating in the National Cancer Institute (NCI) cooperative clinical trials. As part of these visits, the RPC has measured and archived a vast amount of dosimetry data over a wide spectrum of makes and models of linear accelerators (linacs). An important part of the on‐site RPC visit to a participating institution is a review of the institution's electron dosimetry parameters. These parameters include the reference output, percent depth dose data, extended‐distance output, and cone‐ratio (CR) values. Until January 2000, the RPC used the American Association of Physicists in Medicine (AAPM) TG‐21 calibration protocol^1^ and the AAPM TG‐25 report^2^ on electron‐beam dosimetry procedures. All data reported in this work were collected prior to January 2000, before the RPC began using the AAPM TG‐51 calibration protocol.^3^

The RPC has reported previously^4^ ^–^ ^6^ on analyses of dosimetry data measured during on‐site dosimetry review visits. These reports revealed that certain parameters such as depth‐dose factors and wedge/tray factors for a particular nominal energy were consistent to within ±2% (2σ) for most linacs of the same make and model. The observed consistency in dosimetry data has permitted the RPC to prepare compendia of “standard data” consisting of at least five data sets measured for the same make and model of linac using the same accessory design. The work presented in this paper is an extension of the “standard data” concept to electron‐beam CRs. During on‐site review visits, time limitations allow the RPC to measure only a few cone sizes for a limited number of beam energies. Therefore, a significant portion of the data analyzed for this work was clinical data provided by participating institutions in addition to the RPC‐measured data.

## MATERIALS AND METHODS

The data analyzed in this work consisted of measurements in over 1100 electron beams of nominal energies from 4 to 20 MeV produced by 274‐megavoltage therapy units. The cone sizes for which measurements were made ranged from 6 cm×6 cm to 25 cm×25 cm (standard sizes). The linac manufacturers included Varian Associates, Inc. (Palo Alto, CA), Siemens Medical Corp. (Iselin, NJ), and Elekta Oncology Systems Inc. (Norcross, GA). The Varian machines included four linac models: 1800, 2100, 2300, and 2500. The Siemens machines included 5 Mevatron models: MD, KD, 6700 series, 74, and 77. The Elekta/Philips machines included the SL18, SL20, and SL25 models.

The RPC measurements were made in a water phantom that allowed for appropriate side and backscatter using a Farmer‐type 0.6 cm^3^ ion chamber: either model NEL 2571 (NE Technology, Ltd., Berkshire, England) or PTW N23333 (PTW, Freiburg, Germany) and read with a Keithley model 602 electrometer (Keithley Instruments, Inc., Cleveland, OH). The vast majority of the RPC‐measured CRs were for electron energies equal to or less than 12 MeV CRs at nominal source‐to‐skin distance (SSD) were determined in accordance with the AAPM TG‐25 report. For the reference cone size, the depth of maximum dose $\left(d_{\max}\right)$ was determined, and the dose per monitor unit ${\left[(D/\text{MU}\right)}_{\text{ref}}]$ at $d_{\max}$ was measured. For the other cone sizes (CS), the depth of $d_{\max}$ was also determined and the dose per monitor unit ${\left[(D/\text{MU}\right)}_{\text{cs}}]$ was measured. The CR was then calculated as:

(1) $$\text{Cone}\;\text{ratio}={\left(D/\text{MU}\right)}_{\text{cs}}{\left(D/\text{MU}\right)}_{\text{ref}}.$$

As pointed out in the Introduction, the data used for this work consisted of a mix of clinical values provided by participating institutions and RPC measurements performed during the on‐site review visits at those institutions.

Several steps were taken to ensure consistency. All data were normalized to a 10 cm×10 cm cone size. To minimize the spread in cone ratio values resulting from uncertainties in beam energy, the mean incident beam energy $\left({\bar{E}}_{o}\right)$, as measured by the RPC, was used for all the data in this work. Data measured at SSDs other than 100 cm were excluded. Efforts were taken to avoid any possible confusion of data among multiple designs of the same size cone for the same model of linac (e.g., type‐2 and type‐3 cones for Varian machines).

## RESULTS AND DISCUSSION

**Figure 1:**
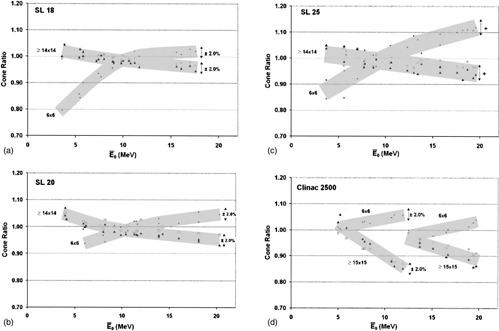
CRs (normalized to 10 cm×10 cm) vs the mean incident energy $\left({\overset{¯}{E}}_{o}\right)$ for 6 cm×6 cm (•), 14 cm×14 cm or 15 cm×15 cm (□), and 20 cm×20 cm (Π) cone sizes. (a) Elekta/Philips SL18, (b) Elekta/Philips SL20, (c) Elekta/Philips SL25, and (d) Varian Clinac 2500.

**Figure 2:**
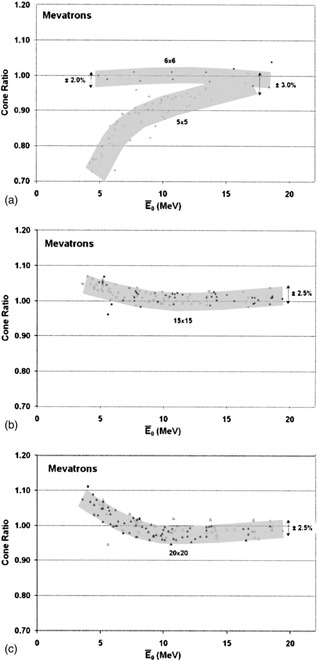
CRs (normalized to 10cm×10cm) vs the mean incident energy $\left({\bar{E}}_{o}\right)$ for Siemens Mevatron machines. (a) 5cm×5cm (people) and 6cm×6cm (●), (b) 15 cm×15 cm (□), and (c) 20cm×20cm (Π).

**Figure 3:**
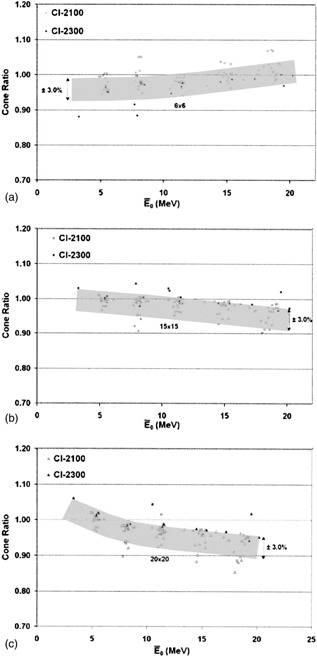
CRs (normalized to 10cm×10cm) versus the mean incident energy $\left({\bar{E}}_{o}\right)$ for Varian machines. (a) 6cm×6cm for Clinac 2100 (people) and Clinac 2300 (●), (b) 15cm×15cm for Clinac 2100 (□), and Clinac 2300 (■), and (c) 20cm×20cm for Clinac 2100 (ρ), and Clinac 2300 (Π).

**Figure 4:**
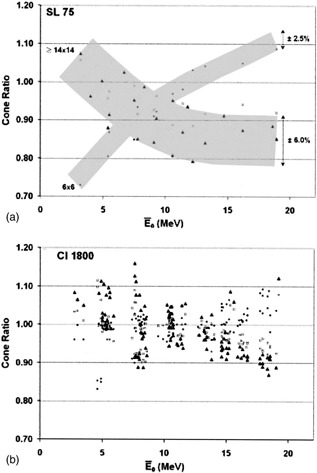
CRs (normalized to 10cm×10cm) versus the mean incident energy $\left({\bar{E}}_{o}\right)$ for 6cm×6cm (●), 14cm×14cm or 15cm×15cm (□), and 20cm×20cm (Π). (a) Elekta/Philips SL75, and (b) Varian Clinac 1800.

All CR data were normalized to the 10 cm×10 cm cone and are presented as a function of incident beam energy $\left({\bar{E}}_{o}\right)$ for each model of linac. The shaded areas in Figs. 1–4 represent cone‐size‐dependent trends. The width of the shaded areas represents two standard deviations (2σ), and therefore includes ~95% of the data pertaining to the trend. The data are tightly clustered in these shaded areas, indicating that the CR values for different linacs of the same model are consistent within the indicated uncertainty of 2σ. With only a couple of exceptions, the 95% confidence interval represents 2% to 3% variation. In other words, the data show that CR values are predictable within the indicated uncertainty for a given model of linac. The RPC refers to consistent data sets such as these as “standard” data and derives representative values from the average of the trend represented by the shaded region.

Figures 1(a) through 1(d) present CR data as a function of mean incident electron energy for four different linac models: the Elekta/Philips SL18, SL20, and SL25 and the Varian Clinac 2500, respectively. Figure 1 demonstrates an almost complete overlap of CR values for the 15cm×15cm (or 14cm×14cm) and 20cm×20cm cone sizes. The 6cm×6cm CRs are distinctively different from the larger cone‐size ratios. In addition, the 6cm×6cm CRs increase with energy, whereas the larger cone‐size ratios decrease with increasing energy. The unusual discontinuity in the trends in Fig. 1(d) is most likely due to a step change in collimator opening at ${\bar{E}}_{o}\sim 12$ MeV. The discontinuity seen in Fig. 1(d) is not observed for the Clinac 2100 and 2300 models as they have a very different head design than the Clinac 2500, which has the target in air, different foil to window distance and different foil to primary jaw difference. Therefore it is not unexpected for the Clinac 2500 data to appear different than the other Clinac data presented. This discontinuity in the trends is not seen for any other linac models presented in this work.

Data are presented that show, in some cases, CR values are consistent not only from one linac to another of the same model but also for different linac models of the same make. Figures 2(a) to 2(c) show the CR trends for the Siemens Mevatron MD, KD, 6700 series, 74, and 77 models for specific cone sizes. The two shaded areas in Fig. 2(a) correspond with 5cm×5cm and 6cm×6cm cone sizes. (Figures 2b) and (2c) show data for 15cm×15cm and 20cm×20cm cone sizes, respectively, where the majority of the data lie within a ±2.5% spread for the five different Mevatron models. The spread (2σ) in the CR data for the five Mevatron models included in Figs. 2(a)–2(c) is 3%. (Figure 2a) includes CRs measured in a 5‐cm diameter circular cone. The larger uncertainty of the data (2σ=3%) is a reflection of the difficulty of measuring dose rate accurately in a small field with a 0.6 cc chamber. Measurements of greater accuracy might be achievable with an instrument having a smaller collecting volume.

Figures 3(a)–3(c) present data for two different linac models manufactured by Varian: the Clinac 2100 series and the Clinac 2300 series. As was the case for the Siemens Mevatron models, these Varian models showed remarkable consistency in CRs. The spread in the data was somewhat greater, being approximately ±3%. This larger spread is attributed to the combining of data measured with both type‐2 and type‐3 electron cones. Although the two cone styles yield slightly different CRs, no attempt was made here to separate the results.

Two of the linac models analyzed demonstrated a spread in CR data that was broader than that presented in the preceding figures. (Figures 4a) and (4b) present CR data for Elekta/Philips SL75 and Varian Clinac 1800 machines, respectively. As shown in (Figure 4a), the data for cone sizes 14cm×14cm and 20cm×20cm overlapped almost perfectly and yet exhibited a large scatter (±~6%). Why this particular linac model showed such a large scatter for these cone sizes is unclear. The Clinac 1800 CR data shown in Fig. 4(b) also demonstrated large variations with no clear trend for any cone size. The manufacturer confirmed that this particular Varian model was provided with only a single‐cone design identified as “Type‐2 accessory” (cones with solid closed walls in contrast to the open‐wall design identified as “Type‐3 accessory”). The variability of the Clinac 1800 CR data can be partly explained by the variability in the primary jaw settings for each cone size between machines. Unlike the Clinac 2100 series of linacs where the jaw positioning is controlled by the software, a resistor in the cone applicator controls the jaw setting which can vary depending on the power supply setting of the linac. The jaw settings can also vary between Clinac 1800s when the flatness and symmetry of the electron beams are being commissioned. Therefore, variability in the jaw settings for each cone size, which strongly influences the cone ratio, will cause a spread in the data shown in Fig. 4(b).

As indicated earlier, a substantial amount of the data presented in Figs. 1–4 were not measured by the RPC. Consequently, the observed scatter in the presented results may result in part from variations in measurement technique among different institutions and possible departures from strict adherence to the TG‐25 recommendations.

## CONCLUSIONS

The analyses presented here show that CR data for many different linacs of the same model bunched together tightly (±2% to 3%), as indicated by the shaded regions of Figs. 1–4. This leads to some important and interesting conclusions.

(i) CR values for a specific cone design are predictable within the stated uncertainty for a given make and model of linac.

(ii) Siemens Mevatron CR values are indistinguishable among the five models: MD, KD, 6700, 74, and 77.

(iii) Among Varian models, CR values are indistinguishable between Clinac 2100C (or 2100 CD) and Clinac 2300C (or 2300 CD).

(iv) Among all Varian machines, the Clinac 2500 is unusual in that it exhibits a discontinuity in CR versus ${\bar{E}}_{o}$ corresponding to 12 MeV.

(v) In general, irrespective of linac model, 6cm×6cm or smaller cones exhibit a monotonic increase in CR values with respect to ${\bar{E}}_{o}$, whereas the opposite behavior is observed for larger cone sizes.

(vi) The RPC “standard” CR values derived as an average from the shaded regions will serve as a quality assurance tool for the RPC to identify potential discrepancies with the clinical values in use at the participating institutions and may serve the physics community as a redundant check for machine commissioning data, clinical data in use, and annual quality assurance measurements.

## ACKNOWLEDGMENTS

This work was supported by Public Health Service Grant No. CA 10953 awarded by the NCI Department of Health and Human Services. The authors would also like to thank the many RPC physicists for assisting in gathering the data as a part of the RPC's quality audit program.

## References

[1] American Association of Physicists in Medicine , “Radiation Therapy Committee Task Group 21: A protocol for determination of absorbed dose from high‐energy photon and electron beams,” Med. Phys. 10, 741–771 (1983). 6419029 10.1118/1.595446

[2] American Association of Physicists in Medicine , “Clinical electron‐beam dosimetry” AAPM Radiation Therapy Committee Task Group 25, Med. Phys. 18, 73–109 (1991). 1901132 10.1118/1.596695

[3] American Association of Physicists in Medicine , “AAPM's TG‐51 protocol for clinical reference dosimetry of high‐energy photon and electron beams,” Med. Phys. 26, 1847–1870 (1999). 10505874 10.1118/1.598691

[4] D. S. Followill , D. S. Davis , and W. F. Hanson , “Standard wedge transmission values for Varian, Siemens, Phillips, and AECL accelerators” Med. Phys. 24, 1076, (1997).

[5] D. S. Davis , D. F. Followill , P. Kennedy , and W. F. Hanson , “Electron percent depth dose and cone ratio from various machines,” Med. Phys. 22, 1007, (1995).

[6] P. M. Kennedy and W. F. Hanson , “A review of high‐energy photon beam characteristics measured by the Radiological Physics Center,” Med. Phys. 19, 838, (1992).

